# Supplementary material for: Life-Course Socioeconomic Trajectories and Biological Aging: The Importance of Lifestyles and Physical Wellbeing
Source: Nutrients. 2024 Oct 2;16(19):3353. doi: 10.3390/nu16193353 (PMC11478881; doi:10.3390/nu16193353)
Supplement: Supplementary file 1 [file nutrients-16-03353-s001.zip › nutrients-3196099-supplementary.pdf]

Supplementary Materials

Blood measurements

Blood samples were obtained between 7 and 9 AM from participants who had fasted overnight and had refrained from smoking for at least 6 hours (h). Biochemical analyses were performed in the centralized Moli-sani laboratory [1]. All hemochromocytometric analyses (white blood cell and platelet counts, granulocyte % and lymphocyte %) were performed by cell counter (Coulter HMX, Beckman Coulter, Milan, Italy) within 3 h from venipuncture. Other details an d full descriptives of blood markers are reported in [2].

Table S1. Components, relevant weights and descriptions of the dietary (DIS) and lifestyle (LIS) inflammatory scores in the Moli-sani study.

Components	Descriptions	Weights
DIS components[1]		
Leafy greens and cruciferous vegetables	Lettuce, broccoli, Brussels sprouts, cauliflower, turnip green, cabbage, kale, and spinach	-0.14
Tomatoes	Tomatoes, and tomato sauce	-0.78
Other vegetables	Peppers, onions, artichokes, celery, mushrooms, eggplant, and beets	-0.16
Apples and berries	Apple, pear, strawberries, and cherry	-0.65
Deep yellow or orange vegetables and fruit	Peach, cooked and row carrots, squash, and figs	-0.57
Other fruits and real fruit juices	Bananas, kiwi, oranges, grapefruits, mandarins, grapefruit, plums, melon, kaki, fruit juices, and orange juices	-0.16
Legumes	Beans, lentils, and chickpeas	-0.04
Fish	Shellfish, shrimps and crustaceous, dried fish (i.e. salted codfish), canned fish and other fish not included in the previous categories (i.e. salmon, swordfish, anchovies, etc.)	-0.08

Poultry	Chicken or turkey with and without skin	-0.45
Red and organ meats	Hamburger, beef, pork, mutton, lamb, and offal	0.02
Processed meats	Canned meat, and processed meat	0.68
High-fat dairy	Whole and semi-skimmed milk, whole milk, fruit yogurt, ice cream and hard cheese.	-0.14
Low-fat dairy	Low-fat cheese, ricotta cheese, and low-fat yogurt.	-0.12
Added sugars	Chocolate, candies, dried fruits, jams, canned fruits, dry cakes, sweetened carbonated beverages, and non-carbonated fruit drinks	0.56
Coffee and tea	Coffee (decaffeinated and caffeinated), and tea	-0.25
Refined grains and starchy vegetables	Breakfast cereals, bread, rice, pasta, potatoes, and home-baked or ready-made cake	0.72
Nuts	Walnuts, hazelnuts, almonds, and peanuts	-0.44
Fats	Butter, gravy, margarine, and mayonnaise or other creamy dressing	0.31
Supplement score[2]	Ranked score of supplements, including: vitamins A, B, C, D, multivitamins, B-complex vitamins and iron	-0.80
LIS components[3]		
Current smoker	Currently smoked tobacco at baseline vs. does not currently smoke tobacco	0.50
Heavy drinker	Heavy [> 1 drink (> 14 g ethanol)/d for women, > 2 drinks (28 g ethanol)/d for men] vs. nondrinker.	0.30

Moderate drinker	Moderate [1 drink (14 g ethanol)/d for women, 1–2 drinks or (14–28 g ethanol)/d for men] vs. nondrinker.	-0.66
Heavily physically active	Exercises ≥ 4 times/week vs. does not exercise	-0.41
Moderately physically active	Exercises 1–3 times/week vs. does not exercise	-0.18
Obese BMI	Obese BMI (≥ 30 kg/m^2) vs. normal/underweight BMI (< 25 kg/m^2)	1.57
Overweight BMI	Overweight BMI (]25–30[kg/m^2) vs. normal/underweight BMI (≤ 25 kg/m^2)	0.89

[1]Dietary components were standardized by sex, to a mean of zero and SD of 1.

[2]All individual supplements were dummy variables, coded as "1" for consumption category and "0" for no-consumption for anti-inflammatory supplements (vitamins A, B, C, D, multivitamins, B-complex vitamins) and "0" or "-1" for pro-inflammatory supplements (iron).

[3]All lifestyle components were dummy variables, coded as 1 for the non-referent category and 0 for the referent category.

Table S2. Comparison of the distribution of SES trajectories between participants included and not included in the analysis.

SES trajectories	Population under study (N= 4,772)	Removed participants (N = 19,553)	P for difference
Stable High	876	3,637	0.73
Education downward	198	740	
Material downward	572	2,407	
Education and material downward	686	2,782	
Education and material upward	422	1,632	
Material upward	283	1,233	
Education upward	278	1,176	
Stable low	1,313	5,273	
Missing	144	673	

P for difference was obtained through a Chi-squared test applied to the counts of each class ($\chi2 = 4.4$, df =7). The number of missing values was not included in the test.

Table S3 Sex-by-SES trajectory interaction analyses.

SES trajectories	$\beta_{interaction}$ (95%CI) (men*SES trajectory)
Stable high	-
Education downward	-1.54 (-3.26 to 0.19)
Material downward	-0.48 (-1.65 to 0.68)
Education and material downward	0.32 (-0.78 to 1.43)
Education and material upward	-0.62 (-1.92 to 0.67)
Material upward	-0.41 (-1.89 to 1.07)
Education upward	-0.23 (-1.73 to 1.27)
Stable low	-0.80 (-1.75 to 0.15)

Regression coefficients (β) with 95% confidence interval (95%CI) for interactive terms with sex ($\beta_{interaction}$ with "men") are reported, from regression models adjusted for chronological age, sex, prevalent CVD, cancer, diabetes, hypertension and hyperlipidemia. Note: For

additive associations with socioeconomic trajectories, see Table 2 in the main text. Sex was not linearly associated with Δage (β (CI) = -0.04 (-0.78 to 0.69)).

Table S4. Sex-stratified association analysis between biological aging (Δage) and SES trajectories.

**SES trajectories**	**β_{men} (95%CI)**<br>**N = 2,299**	**β_{women} (95%CI)**<br>**N = 2,473**
Stable high	-	-
Education downward	-0.23 (-1.45 to 1.00)	1.29 (0.08 to 2.51)
Material downward	0.03 (-0.83 to 0.88)	0.57 (-0.22 to 1.37)
**Education and material downward**	**1.42 (0.61 to 2.22)**	**1.08 (0.31 to 1.84)**

Education and material		
upward	0.04 (-0.88 to 0.96)	0.57 (-0.37 to 1.51)
Material upward	0.46 (-0.64 to 1.55)	0.72 (-0.33 to 1.78)
Education upward	0.35 (-0.71 to 1.41)	0.56 (-0.49 to 1.62)
**Stable low**	0.35 (-0.39 to 1.08)	**1.02 (0.34 to 1.70)**

Regression coefficients (95%CI) obtained from a model controlled for chronological age, prevalent CVD, cancer, diabetes, hypertension and hyperlipidemia. Significant associations ($p < 0.05$) are highlighted in bold.

Figure S1. Flowchart for the selection of study participants from the Moli-sani Study.

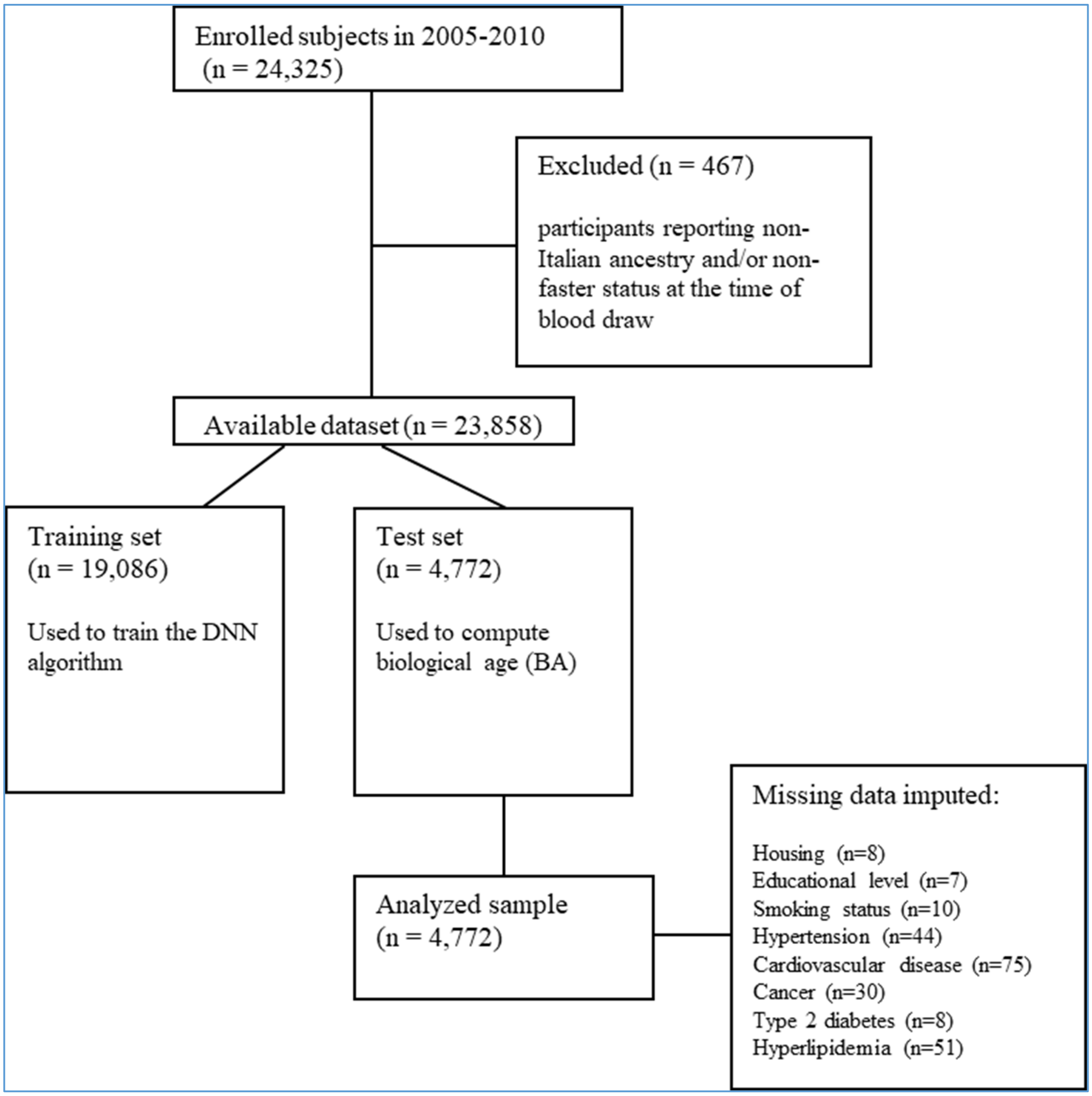

Abbreviations: DNN = deep neural network.

References

1. Santimone, I.; Di Castelnuovo, A.; De Curtis, A.; Spinelli, M.; Cugino, D.; Gianfagna, F.; Zito, F.; Donati, M.B.; Cerletti, C.; de Gaetano, G.; et al. White blood cell count, sex and age are major determinants of heterogeneity of platelet indices in an adult general population: results from the MOLI-SANI project. *Haematologica* **2011**, *96*, 1180–1188, doi:10.3324/haematol.2011.043042.
2. Gialluisi, A.; Di Castelnuovo, A.; Costanzo, S.; Bonaccio, M.; Persichillo, M.; Magnacca, S.; De Curtis, A.; Cerletti, C.; Donati, M.B.; de Gaetano, G.; et al. Exploring domains, clinical implications and environmental associations of a deep learning marker of biological ageing. *Eur. J. Epidemiol.* **2021**, doi:10.1007/s10654-021-00797-7.

Moli-sani Study Investigators

The enrolment phase of the Moli-sani Study was conducted at the Research Laboratories of the Catholic University in Campobasso (Italy), the follow up of the Moli-sani cohort is being conducted at the Department of Epidemiology and Prevention of the IRCCS Neuromed, Pozzilli, Italy.

Steering Committee: Licia Iacoviello*°(Chairperson), Giovanni de Gaetano* and Maria Benedetta Donati*.

Scientific Secretariat: Marialaura Bonaccio*, Americo Bonanni*, Chiara Cerletti*, Simona Costanzo*, Amalia De Curtis*, Augusto Di Castelnuovo§, Alessandro Gialluisi*°, Francesco Gianfagna°§, Mariarosaria Persichillo*, Teresa Di Prospero* (Secretary).

Safety and Ethical Committee: Jos Vermylen (Catholic University, Leuven, Belgio) (Chairperson), Renzo Pegoraro (Pontificia Accademia per la Vita, Roma, Italy), Antonio Spagnolo (Catholic University, Roma, Italy).

External Event Adjudicating Committee: Deodato Assanelli (Brescia, Italy), Livia Rago (Campobasso, Italy).

Baseline and Follow-up Data Management: Simona Costanzo* (Coordinator), Marco Olivieri (Campobasso, Italy), Teresa Panzera*.

Data Analysis: Augusto Di Castelnuovo§ (Coordinator), Marialaura Bonaccio*, Simona Costanzo*, Simona Esposito*, Alessandro Gialluisi*°, Francesco Gianfagna°§, Sabatino Orlandi*, Emilia Ruggiero*, Alfonsina Tirozzi*.

Biobank, Molecular and Genetic Laboratory: Amalia De Curtis* (Coordinator), Sara Magnacca§, Fabrizia Noro*, Alfonsina Tirozzi*.

Recruitment Staff: Mariarosaria Persichillo* (Coordinator), Francesca Bracone*, Teresa Panzera*.

Communication and Press Office: Americo Bonanni*.

Regional Institutions: Direzione Generale per la Salute - Regione Molise; Azienda Sanitaria Regionale del Molise (ASReM, Italy); Agenzia Regionale per la Protezione Ambientale del Molise (ARPA Molise, Italy); Molise Dati Spa (Campobasso, Italy); Offices of vital statistics of the Molise region.

Hospitals: Presidi Ospedalieri ASReM: Ospedale A. Cardarelli – Campobasso, Ospedale F. Veneziale – Isernia, Ospedale San Timoteo - Termoli (CB), Ospedale Ss. Rosario - Venafro (IS), Ospedale Vietri – Larino (CB), Ospedale San Francesco Caracciolo - Agnone (IS); Casa di Cura Villa Maria - Campobasso; Ospedale Gemelli Molise - Campobasso; IRCCS Neuromed - Pozzilli (IS).

*Department of Epidemiology and Prevention, IRCCS Neuromed, Pozzilli, Italy
°Department of Medicine and Surgery, University of Insubria, Varese, Italy
§Mediterranea Cardiocentro, Napoli, Italy

Moli-sani Study Past Investigators are available at https://www.moli-sani.org/?page_id=173
